# A programmed wave of uridylation-primed mRNA degradation is essential for meiotic progression and mammalian spermatogenesis

**DOI:** 10.1038/s41422-018-0128-1

**Published:** 2019-01-07

**Authors:** Marcos Morgan, Yuka Kabayama, Christian Much, Ivayla Ivanova, Monica Di Giacomo, Tatsiana Auchynnikava, Jack Michael Monahan, Dimitrios Michael Vitsios, Lina Vasiliauskaitė, Stefano Comazzetto, Juri Rappsilber, Robin Campbell Allshire, Bo Torben Porse, Anton James Enright, Dónal O’Carroll

**Affiliations:** 1 MRC Centre for Regenerative Medicine, School of Biological Sciences, University of Edinburgh, 5 Little France Drive, Edinburgh, EH16 4UU UK; 2 European Molecular Biology Laboratory (EMBL), Via Ramarini 32, 00015 Monterotondo, Italy; 3 Wellcome Centre for Cell Biology, University of Edinburgh, Edinburgh, EH9 3BF UK; 4 European Bioinformatics Institute, Hinxton, Cambridge CB10 1SD UK; 5 Institute of Biotechnology, Technische Universität Berlin, Berlin, 13355 Germany; 6 Biotech Research and Innovation Center (BRIC), University of Copenhagen, Copenhagen, 2200 Denmark; 7 The Finsen Laboratory, Rigshospitalet, Faculty of Health Sciences, University of Copenhagen, Copenhagen, 2200 Denmark; 8 Danish Stem Cell Centre (DanStem), Faculty of Health Sciences, University of Copenhagen, Copenhagen, 2200 Denmark

**Keywords:** RNA modification, Developmental biology

## Abstract

Several developmental stages of spermatogenesis are transcriptionally quiescent which presents major challenges associated with the regulation of gene expression. Here we identify that the zygotene to pachytene transition is not only associated with the resumption of transcription but also a wave of programmed mRNA degradation that is essential for meiotic progression. We explored whether terminal uridydyl transferase 4- (TUT4-) or TUT7-mediated 3′ mRNA uridylation contributes to this wave of mRNA degradation during pachynema. Indeed, both TUT4 and TUT7 are expressed throughout most of spermatogenesis, however, loss of either TUT4 or TUT7 does not have any major impact upon spermatogenesis. Combined TUT4 and TUT7 (TUT4/7) deficiency results in embryonic growth defects, while conditional gene targeting revealed an essential role for TUT4/7 in pachytene progression. Loss of TUT4/7 results in the reduction of miRNA, piRNA and mRNA 3′ uridylation. Although this reduction does not greatly alter miRNA or piRNA expression, TUT4/7-mediated uridylation is required for the clearance of many zygotene-expressed transcripts in pachytene cells. We find that TUT4/7-regulated transcripts in pachytene spermatocytes are characterized by having long 3′ UTRs with length-adjusted enrichment for AU-rich elements. We also observed these features in TUT4/7-regulated maternal transcripts whose dosage was recently shown to be essential for sculpting a functional maternal transcriptome and meiosis. Therefore, mRNA 3′ uridylation is a critical determinant of both male and female germline transcriptomes. In conclusion, we have identified a novel requirement for 3′ uridylation-programmed zygotene mRNA clearance in pachytene spermatocytes that is essential for male meiotic progression.

## Introduction

Spermatogenesis is the developmental process that produces spermatozoa and underpins male fertility throughout adult life. There are three distinct phases of spermatogenesis: the mitotic, meiotic, and spermiogenic stages that each presents distinct cellular challenges. Spermatogonia are the mitotic germ cells encompassing the spermatogonial stem cells, which maintain spermatogenesis throughout adulthood, and the transient amplifying and differentiating spermatogonial populations, which divide prolifically to expand the pool of cells that will enter into meiosis and ultimately complete spermatogenesis.^1,2^ During meiosis one round of DNA replication, meiotic recombination, and two successive cell divisions produce haploid spermatids containing recombinant chromosomes. The process of meiotic chromosome recombination is associated with distinct transcriptional and chromatin alterations that present several challenges to the cell. The early leptotene and zygotene stages of meiosis when chromosomes align and pair with the assembly of the synaptonemal complex, are transcriptionally inert.^3,4^ Transcriptional resumption occurs at the pachytene stage, when meiotic recombination also occurs, and is associated with major changes to the chromatin template^5–7^ that result in pervasive genomic transcription, including LINE1 and distinct ERV^8,9^ transposons. Spermiogenesis is the final stage of spermatogenesis where round spermatids terminally differentiate to become spermatozoa. This process is also associated with dramatic changes to the chromatin landscape that accompany the condensation and repackaging of the genome where histones are ultimately replaced by protamines in spermatozoa.

The varying transcriptional status during spermatogenesis necessitates post-transcriptional regulation of spermatogenic transcriptomes. Indeed, several RNA degradation and modification pathways are acutely important for male germ cell development. The mRNA modification m^6^A is required for spermatogenesis^10–13^ with the m^6^A-reader YTH domain-containing 2 (YTHDC2) required to repress mitotic transcripts for cells to be able to enter/complete meiosis.^14–17^ The RNA-modification m^5^C is required for pachytene progression but the mechanism by which m^5^C functions therein is not understood.^18^ piRNAs post-transcriptionally silence LINE1 elements by directing PIWI protein-mediated endonucleolytic cleavage of LINE1 transcripts in pachytene and round spermatid transcriptomes.^8,19^ piRNAs also function in transcriptome clearance during spermiogenesis.^19–23^ The miRNA pathway is also essential for spermatogenesis^24–27^ with the miR-34 family of miRNAs regulating both pachytene and elongating spermatid transcriptomes.^24,28–31^ Thus the resumption of transcription in pachytene spermatocytes requires at least two small non-coding RNA pathways. However, whether other mRNA degradation pathways or RNA modifications are required for meiotic progression remains unknown.

Non-templated mRNA 3′ uridylation plays important roles in miRNA biogenesis as well as in mRNA turnover. TUT4 and TUT7 (TUT4/7) are the main cellular terminal uridylyltransferases that mediate miRNA and mRNA uridylation.^32,34,35^ However, whether 3′ ends of piRNAs can be uridylated by TUT4/7 remains unknown. TUT4/7-mediated precursor-miRNA (pre-miRNA) 3′ uridylation participates in both the positive and negative regulation of miRNA biogenesis.^34,36–39^ LIN28a recruits TUT4/7 to pre-let-7 where subsequent oligo-uridylation targets the pre-miRNAs for degradation through the recruitment of DIS3L2,^40,41^ a 3′–5′ ribonuclease that specifically recognizes RNA with an oligo(U) tail.^40–43^ In cells where LIN28a is not expressed, TUT4/7-mediated terminal mono-uridylation of a small group of pre-miRNAs optimizes their biogenesis through the generation of a two-nucleotide overhang, which is the favored substrate of DICER.^37^ Deadenylation is a key determinant of mRNA turnover that can be augmented by TUT4/7-mediated 3′ uridylation.^32,44^ Transcripts in which the poly(A) tails are shortened to less than ~27 nucleotides show subsequent loss of the stabilizing poly-A binding proteins (PABPs) that in turn facilitates TUT4/7-mediated 3′ uridylation.^32^ This uridylation optimizes transcripts for decay through the recruitment of LSM (like Sm) proteins,^32,45^ which engages the DCP1/2 mRNA decapping enzymes and ultimately the 5′–3′ XRN1-mediated decay pathway.^46^

Interestingly, TUT4/7-mediated mRNA 3′ uridylation is required for the maternal to zygotic transition in several vertebrate species and may constitute an innate viral restriction mechanism conserved from *C. elegans* to humans.^47,48^ While a variety of somatic mouse cell lines and tissues are not dependent upon TUT4/7-mediated mRNA 3′ uridylation for mRNA degradation, it is essential for the formation of the mouse maternal transcriptome by targeting a variety of transcripts for degradation during oocyte growth.^44^ Here, we have sought to determine whether TUT4/7-mediated mRNA 3′ uridylation is a common requirement for germ cell development.

## Results

### A major wave of mRNA degradation occurs in pachytene spermatocytes

**Fig. 1:**
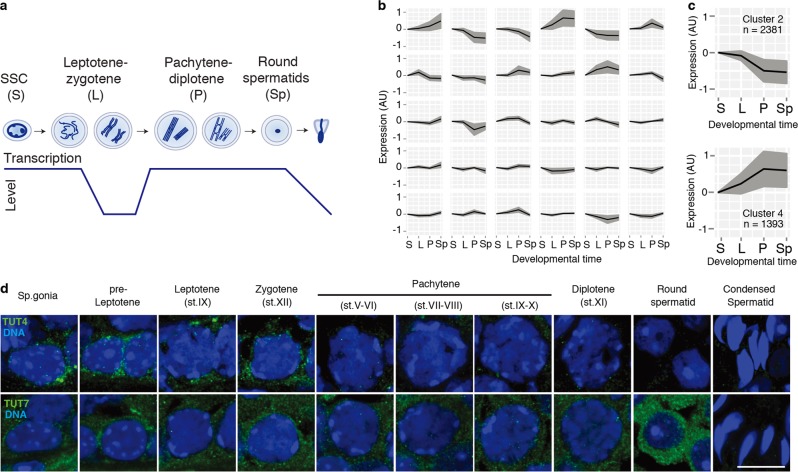
A programmed wave of RNA degradation takes place during the leptotene-zygotene to pachytene transition. **a** Schematic representation of different spermatogenic transitions. Spermatogonial stem cells (SSC; S), leptotene-zygotene (L), pachytene-diplotene (P), and round spermatids (Sp). Levels of transcriptional activity are indicated below. **b** Expression change across different spermatogenic transitions as defined in **a** for clusters of transcripts with similar expression profiles. Clusters were generated using the Markov clustering algorithm. The black line indicates the mean expression of the group, and the gray area indicates the standard deviation. **c** Expression changes as defined in **b** for the two clusters showing changes in gene expression during the leptotene-zygotene to pachytene transition (*n*, number of genes). **d** Confocal immunofluorescence micrographs of different spermatogenic cell types are shown. Testes from WT animals were stained with an antibody against TUT4 (green), upper panel. Testes from *Tut7*^*HA-GFP/HA-GFP*^ mice were stained with an anti-HA antibody (green), lower panel. DNA was stained with Hoechst 33342 (blue). Scale bar, 10 μm

The dynamic changes to chromosome, DNA, chromatin, and transcription during spermatogenesis result in many challenges for male germ cells. One such challenge is the necessity to post-transcriptionally regulate the transcriptome due to periods of transcriptional quiescence. We sought to understand at which stage the major changes in the transcriptome occur during spermatogenesis. To this end, we profiled gene expression from mitotic spermatogonial stem cells, meiotic leptotene-zygotene cells, meiotic pachytene cells, and post-meiotic round spermatids (Fig. 1a). We then applied the Markov clustering algorithm to identify gene expression patterns across spermatogenesis (Fig. 1b). Two prominent clusters were identified by massive changes in gene expression during meiosis between leptotene-zygotene and pachytene spermatocytes coinciding with the resumption of transcription as cells enter pachytene (Fig. 1c). Cluster 4 (1393 genes) represents a sharp upregulation of transcripts in pachytene that can be rationalized by their transcription. Cluster 2 (2381 genes) corresponds to genes that are abruptly downregulated as cells enter pachytene; this requires a large component of RNA degradation to rationalize the decreased dosage of these transcripts given that leptotene-zygotene cells are transcriptionally inert. This pattern was also identified using an independent higher resolution single-cell dataset^49^ (Fig. S1). Given the prominent role for the terminal uridylyltransferases TUT4 and TUT7 in sculpting the maternal transcriptome,^44^ we hypothesized that 3′ mRNA uridylation may also contribute to post-transcriptional regulation during spermatogenesis. Both TUT4 and TUT7 are expressed throughout spermatogenesis, despite that the expression of TUT7 is upregulated in round spermatids whereas TUT4 expression is downregulated during meiosis (Fig. 1d). Therefore, it is possible that mRNA 3′ uridylation could play a role in the regulation of the spermatogenic transcriptomes.

### Loss of TUT4 or TUT7 alone does not have an impact upon male fertility, but combined TUT4/7 deficiency results in perinatal lethality

**Fig. 2:**
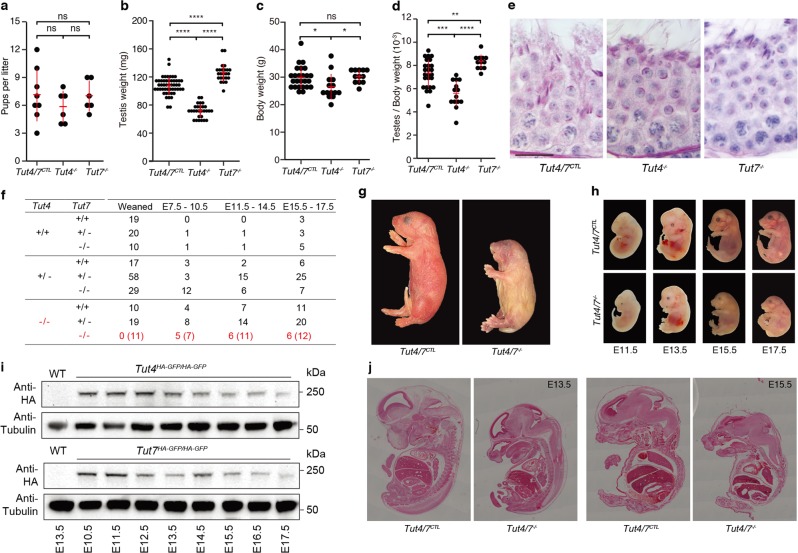
TUT4-deficient and TUT7-deficient animals are fertile but combined TUT4/7-deficient animals show growth retardation and die perinatally. **a** Pups per litter for *Tut4/7*^*CTL*^, *Tut4*^*−/*−^, and *Tut7*^*−/−*^ males are shown. Each dot corresponds to a single litter. The center value represents the mean, and the error bars the standard deviation. (ns not significant; Wilcoxon test two-sided; *Tut4/7*^*CTL*^, litters = 8, sires = 3; *Tut4*^*−/−*^, litters = 6, sires = 3; *Tut7*^−/−^, litters = 6, sires = 3). **b** Testis weight for *Tut4/7*^*CTL*^, *Tut4*^*−/−*^, and *Tut7*^−*/–*^ animals. Each dot corresponds to a single testis. The center value represents the mean, and the error bars the standard deviation. (****P *<* 0.0001, Wilcoxon test two-sided; *Tut4/7*^*CTL*^, *n* = 50; *Tut4*^−*/*−^, *n* = 28; *Tut7*^*−/−*^, *n* = 24). **c** Body weight for *Tut4/7*^*CTL*^, *Tut4*^−/−^, and *Tut7*^−/−^ males. Each dot corresponds to the body weight of a single animal. The center value represents the mean, and the error bars the standard deviation. (ns not significant; *P < 0.05, Wilcoxon test two-sided; *Tut4/7*^*CTL*^, *n* = 25; *Tut4*^−/−^, *n* = 14; *Tut7*^−*/*−^, *n* = 12). **d** Ratio of testes to body weight for *Tut4/7*^*CTL*^, *Tut4*^−/−^, and *Tut7*^−/−^ males. Each dot corresponds to the testes/body weight ratio of a single animal. The center value represents the mean, and the error bars the standard deviation. (**P < 0.05, ***P < 0.01, ****P < 0.001, Wilcoxon test two-sided). **e** Micrographs of PAS-stained tubule sections from *Tut4/7*^*CTL*^, *Tut4*^*−/*−^, and *Tut7*^*−/−*^ mice. Scale bar, 20 μm. **f** Table of pups/embryos observed for different genotypes at different stages of development (weaned, E7.5-10.5, E11.5-14.5, and E15.5-E17.5). The expected numbers of TUT4/7-deficient animals are shown in brackets. **g** Pictures of P0 *Tut4/7*^*CTL*^ and *Tut4/7*^*−/*−^ animals are shown. Objective magnification, 1 × . **h** Pictures of *Tut4/7*^*CTL*^ and *Tut4/7*^−*/*−^ mice at embryonic stages E11.5, E13.5, E15.5, and E17.5 are shown. Objective magnification, 2× for E11.5 and E13.5 embryos and 1× for E15.5 and E17.5 embryos. **i** Western blots of extracts from WT, *Tut4*^*HA-GFP/HA-GFP*^ *and Tut7*^*HA-GFP/HA-GFP*^ embryos at different stages of development (E10.5 to E17.5) using anti-HA and anti-Tubulin antibodies. **j** H&E stained sections of *Tut4/7*^*CTL*^ and *Tut4/7*^−/−^ animals at E13.5 and E15.5 embryonic stages are shown. Objective magnification, 10×

To understand the contribution of TUT4- and TUT7-mediated uridylation to spermatogenesis, we analyzed *Tut4*^−*/*−^ and *Tut7*^−*/*−^ mice. While both *Tut4*^*−/*−^ and *Tut7*^*−/*−^ male mice were fertile (Fig. 2a), *Tut4*^*−/*−^ testes were reduced in mass (Fig. 2b), partially accounted for by reduced body mass (Fig. 2c, d). Histological analysis revealed normal spermatogenesis in the seminiferous tubules of both genotypes (Fig. 2e) although a small fraction (~10%) of seminiferous tubules with aberrant spermatogenesis was observed in *Tut4*^*−/−*^ testes (Fig. S2). The mild defects in *Tut4*^*−/*−^ testes suggest functional redundancy between TUT4 and TUT7. To test this possibility, we established crosses to derive *Tut4*^*−/−*^*; Tut7*^−*/*−^ mice, however no *Tut4*^*−/*−^*; Tut7*^*−/−*^ pups were observed at weaning (Fig. 2f). Mice are nocturnal animals and usually give birth at night; pups that die perinatally are often eaten by the mother.^50^ The morning after birth, we occasionally retrieved severely growth retarded *Tut4*^*−/−*^*; Tut7*^*−/*−^ dead mice, indicating that combined TUT4/7-deficiency results in perinatal lethality (Fig. 2g). We next sought to understand the impact of TUT4/7 ablation on embryonic development; *Tut4*^*−/*−^*; Tut7*^*−/*−^ embryos were retrieved in approximately half the expected numbers from mid through late gestation (Fig. 2f). Furthermore, growth retardation was observed from E11.5 that became progressively worse through gestation (Fig. 2h). These results are consistent with the fact that both TUT4 and TUT7 are expressed throughout mid to late embryonic development (Fig. 2i). Histological analysis revealed that the embryonic morphogenesis was normal (Fig. 2j) indicating that *Tut4*^−*/*−^*; Tut7*^−/−^ embryos suffered from defective growth that is incompatible with viability. In summary, TUT4 and TUT7 are functionally redundant and required for embryonic growth.

### TUT4 and TUT7 and their uridylation activity are essential for male meiosis

**Fig. 3:**
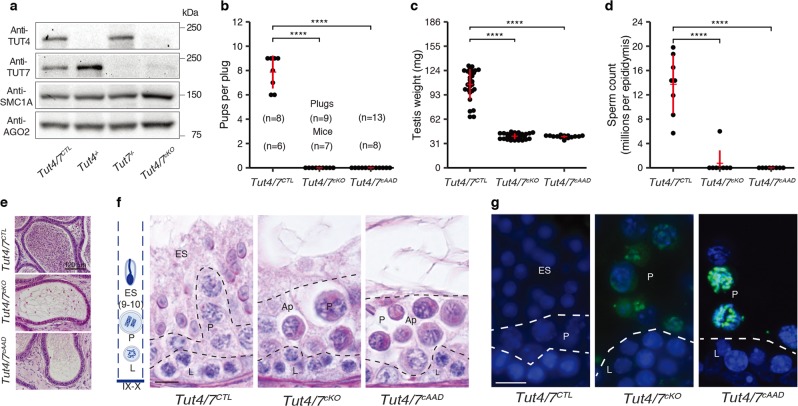
TUT4/7 and their uridylation activity are required for male fertility and pachytene progression. **a** Western blots from whole testes extracts of *Tut4/7*^*CTL*^, *Tut4*^*−/−*^, *Tut7*^*−/−*^, and *Tut4/7*^*cKO*^ animals using anti-TUT4, TUT7, AGO2 and SMC1A antibodies. **b** Pups per plug for *Tut4/7*^*CTL*^, *Tut4/7*^*cKO*^, and *Tut4/7*^*cAAD*^ males mated with WT females. The numbers of plugs and sires for each group are indicated. Each dot represents a single litter size. The mean, and standard deviations are indicated in red, together with the groups’ difference significance (****P < 0.001, Wilcoxon test two-sided). **c** Testis weight of *Tut4/7*^*CTL*^, *Tut4/7*^*cKO*^ and *Tut4/7*^*cAAD*^ mice. Each dot corresponds to a single testis. The center value represents the mean, and the error bars the standard deviation (****P < 0.001, Wilcoxon test two-sided; *Tut4/7*^*CTL*^, *n* = 28; *Tut4/7*^*cKO*^, *n* = 28; *Tut4/7*^*cAAD*^, *n* = 15). **d** Sperm count per epididymis of *Tut4/7*^*CTL*^, *Tut4/7*^*cKO*^, and *Tut4/7*^*cAAD*^ animals. Each dot corresponds to the sperm count from a single epididymis. The center value represents the mean, and the error bars the standard deviation (****P < 0.001, Wilcoxon test two-sided; *Tut4/7*^*CTL*^, *n* = 8; *Tut4/7*^*cKO*^, *n* = 8; *Tut4/7*^*cAAD*^, *n* *=* 8). **e** Sections of epididymis from *Tut4/7*^*CTL*^, *Tut4/7*^*cKO*^, and *Tut4/7*^*cAAD*^ animals stained with Hematoxylin and Eosin. Scale bar, 120 μm. **f** Sections of stage IX-X tubules from *Tut4/7*^*CTL*^, *Tut4/7*^*cKO*^, and *Tut4/7*^*cAAD*^ mice stained with the PAS method. The different cell layers are indicated: stages IX-X elongated spermatids (ES), pachytene (P), leptotene (L). Apoptotic cells are also indicated (Ap). On the left, a schematic representation of the different layers found in a stage IX-X tubule is shown. Scale bar, 10 μm. **g** Tubule sections from *Tut4/7*^*CTL*^, *Tut4/7*^*cKO*^, and *Tut4/7*^*cAAD*^ animals stained with TUNEL. DNA was stained with Hoechst 33342 (blue). Scale bar, 10 μm. Cell types are indicated as in **f**

The perinatal lethality of *Tut4*^*−/−*^*; Tut7*^*−/−*^ mice necessitates the use of conditional genetics to understand their contribution to spermatogenesis. We thus combined *Tutf4*^*fl*^ and *Tut7*^*fl*^ alleles^44^ with a *Stra8Cre* transgene that induces gene deletion as cells enter meiosis^51,52^ to generate control *Tut4*^*+/fl*^*; Tut7*^*+/fl*^*; Stra8Cre Tg*^*+*^ *or Tut4*^*+/+*^*; Tut7*^*+/+*^*; Stra8Cre Tg*^*+*^ (*Tut4/7*^*CTL*^) and experimental *Tut4*^*fl/fl*^*; Tut7*^*fl/fl*^*; Stra8Cre Tg*^*+*^ (*Tut4/7*^*cKO*^) mice. This strategy resulted in the conditional ablation of both TUT4 and TUT7 (Fig. 3a). *Tut4/7*^*cKO*^ mice were infertile when crossed to wild-type female mice (Fig. 3b). Atrophic testis in *Tut4/7*^*cKO*^ mice accompanied by loss of spermatozoa suggested spermatogenic failure in *Tut4/7*^*cKO*^ mice (Fig. 3c–e). Histological analysis as well as γH2AX and SCP3 staining of *Tut4/7*^*cKO*^ testes revealed spermatogenic arrest in late pachytene (Stages IX-X) accompanied by apoptosis (Fig. 3f, g and Figs. S3–4). To understand whether the spermatogenesis phenotype in *Tut4/7*^*cKO*^ mice is dependent upon the TUT4/7-mediated uridylation, we took advantage of the *Tut4*^*AAD*^ allele^44^ in which mutations in the catalytic triad (DDD to AAD) renders TUT4 enzymatically inactive.^38,53^ We combined the *Tut4*^*fl*^*, Tut7*^*fl*^, *Tut4*^*AAD*^, and *Stra8Cre* alleles to generate experimental *Tut4*^*fl/AAD*^*; Tut7*^*fl/fl*^*; Stra8Cre Tg*^*+*^ (*Tut4/7*^*cAAD*^) mice, where only a single copy of the catalytic-dead TUT4^AAD^ protein is expressed as cells enter meiosis. The expression of the TUT4^AAD^ protein resulted in the exact same phenotype as observed in mice with conditional deletion of TUT4/7 (Fig. 3a–g and Figs. S3–4), whereas a single copy of TUT4 supported spermatogenesis. In summary, TUT4/7 and specifically their uridylation activity autonomously underpin male meiosis.

**Fig. 4:**
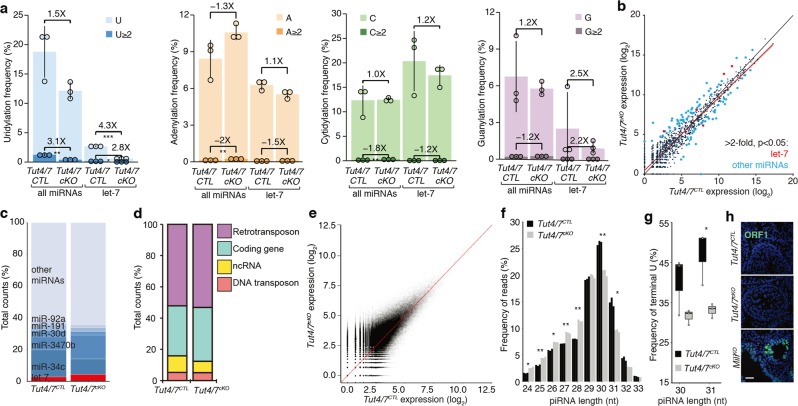
TUT4/7-deficiency has a minor impact on pachytene small RNA pathways. **a** Frequency of 3′ terminal uridylation (blue), adenylation (orange), cytidylation (green), and guanylation (purple) for all pachytene miRNAs or let-7 miRNAs from *Tut4/7*^*CTL*^ and *Tut4/7*^*cKO*^ animals. For each group, the frequency of mono- (light) and oligo-nucleotide additions (dark) is shown. The fold change between the different groups is indicated together with the significance (*P < 0.05, **P < 0.01, ***P < 0.001, *t*-test two-sided; *Tut4/7*^*CTL*^, *n* = 3; *Tut4/7*^*cKO*^, *n* = 3). Each dot represents a biological replicate. The heights of the bars indicate the mean value for the different replicates, and the error bars show the standard deviation. **b** Scatter plot of miRNA expression levels in *Tut4/7*^*CTL*^ vs *Tut4/7*^*cKO*^ pachytene cells. Let-7 miRNA significantly changing more than two-fold (*P* < 0.05, Wald test) are highlighted in red. Other miRNAs significantly changing more than two-fold (*P* < 0.05, Wald test) are highlighted in blue. (*Tut4/7*^*CTL*^, *n* = 3; *Tut4/7*^*cKO*^, *n* = 3). The linear regression and the identity line are indicated in red and black, respectively. **c** Read count frequency of different miRNA families from *Tut4/7*^*CTL*^ and *Tut4/7*^*cKO*^ pachytene spermatocytes. **d** Read count frequency of different piRNA deriving from retrotransposons, DNA transposons, coding genes, and other non-coding RNAs (ncRNA) in *Tut4/7*^*CTL*^ and *Tut4/7*^*cKO*^ pachytene cells. **e** Scatter plot of all piRNA expression levels in *Tut4/7*^*CTL*^ vs *Tut4/7*^*cKO*^ pachytene spermatocytes. The identity line is shown in red. **f** Normalized length distribution of pachytene piRNA from *Tut4/7*^*CTL*^ and *Tut4/7*^*cKO*^ males. Each bar corresponds to a biological replicate. (*P < 0.05, **P < 0.01, *t*-test two-sided; *Tut4/7*^*CTL*^, *n* = 3; *Tut4/7*^*cKO*^, *n* = 3). **g** Terminal U frequency for 30 and 31 nucleotide long pachytene piRNAs from *Tut4/7*^*CTL*^ and *Tut4/7*^*cKO*^ males. Each dot corresponds to a biological replicate. The center value represents the median, and the error bars the range (*P < 0.05, *t*-test one-sided; *Tut4/7*^*CTL*^, *n* = 3; *Tut4/7*^*cKO*^, *n* = 3). **h** Confocal micrographs of testes sections from *Tut4/7*^*CTL*^, *Tut4/7*^*cKO*^, and *Mili*^*KO*^ animals stained with an anti-ORF1 LINE1 antibody (green) are shown. DNA was stained with Hoechst 33342 (blue). Scale bar, 40 μm

### The loss of TUT4/7 does not greatly affect pachytene small RNA pathways

Progression through meiosis is dependent upon Argonaute-bound small RNA (sRNA) pathways in male mice. Both the microRNA (miRNA) and PIWI-interacting (piRNA) pathways are essential for normal pachytene progression.^8,24,25,54^ TUT4 and TUT7 are *bona fide* regulators of miRNAs whereas a role for 3′ uridylation in the piRNA pathway remains unknown. We, therefore, performed sRNA-seq on RNA isolated from sorted pachytene cells. The analysis of miRNA identified a reduction in terminal miRNA mono- and oligo-uridylation that was most pronounced in the let-7 miRNA family (Fig. 4a). The loss of TUT4/7 did not have an impact upon miRNA terminal guanylation, adenylation or cytidylation (Fig. 4a). The reduction of 3′ uridylation in *Tut4/7*^*cKO*^ pachytene cells did not grossly alter miRNA expression (Fig. 4b, c). The expression of the top five most abundantly expressed miRNAs remains almost unchanged (Fig. 4c), as well as that of other miRNAs previously implicated in spermatogenesis^55^ (Fig. S5). As expected, most let-7 miRNAs are modestly upregulated in the absence of TUT4/7. Similar changes in miRNA expression were observed in MEFs, ESCs, liver and bone marrow from *Tut4/7*^*iKO*^ mice and these changes did not result in the alteration of the mRNA transcriptome.^44^ We thus conclude that the differences observed in miRNA expression in *Tut4/7*^*cKO*^ pachytene cells are unlikely to contribute to the observed pachytene arrest.

Analysis of pachytene piRNAs from *Tut4/7*^*CTL*^ and *Tut4/7*^*cKO*^ pachytene spermatocytes did not reveal differences in piRNA identity, frequency, or annotation (Fig. 4d, e). We did, however, observe a higher frequency of long piRNAs comprising 30 and 31 nucleotides in *Tut4/7*^*CTL*^ spermatocytes (Fig. 4f). Analysis of these piRNAs revealed a marked decrease in the frequency of 3′ uridine in *Tut4/7*^*cKO*^ pachytene spermatocytes (Fig. 4g), indicating that 30–31 nucleotide long piRNAs can be uridylated by TUT4/7. One critical and measurable meiotic piRNA function is to guide the PIWI protein MILI to endonucleolytically cleave LINE1 transcripts. Failure to do so results in deregulated LINE1 expression, DNA damage, and spermatogenic arrest.^8,56^ LINE1 elements were appropriately silenced in *Tut4/7*^*cKO*^ seminiferous tubules (Fig. 4h); thus, the loss of TUT4/7 does not impact piRNA dosage nor function.

### TUT4/7-mediated mRNA 3′ uridylation eliminates transcripts in pachytene spermatocytes

**Fig. 5:**
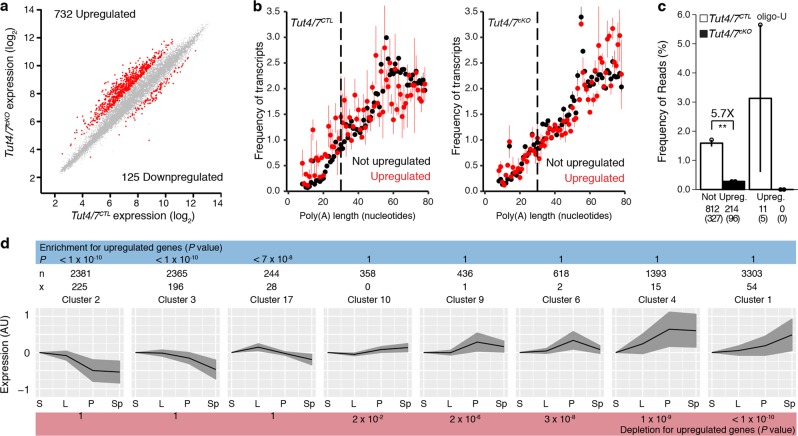
TUT4/7 are required for clearance of transcripts in pachytene spermatocytes. **a** Scatter plot of pachytene mRNA expression levels in *Tut4/7*^*CTL*^ vs *Tut4/7*^*cKO*^ animals. Transcripts significantly changed (*P* < 0.01 and fold-change >2, Moderated t-statistic adjusted; *Tut4/7*^*CTL*^, *n* = 4; *Tut4/7*^*cKO*^, *n* = 3) are highlighted in red. The numbers of significantly upregulated and downregulated genes are indicated. **b** Poly(A) tail length distribution for pachytene mRNAs from *Tut4/7*^*CTL*^ and *Tut4/7*^*cKO*^ mice. The distribution is shown for upregulated (red) or not upregulated (black) transcripts. Each dot represents the mean value of two biological replicates and the error bars indicate the range. The dotted vertical line at 30 nucleotides separates short and long tails. **c** Oligo-uridylation of pachytene mRNAs with short poly(A) tails from *Tut4/7*^*CTL*^ and *Tut4/7*^*cKO*^ mice. Each dot represents a biological replicate. The height of the bar and the error bars represent the mean and range, respectively. The fold change between groups is indicated together with its significance (**P < 0.01, *t*-test two-sided; *Tut4/7*^*CTL*^, *n* = 2; *Tut4/7*^*cKO*^, *n* = 2). The number of transcripts and genes (in brackets) is shown for each group. **d** Enrichment analysis of upregulated transcripts in *Tut4/7*^*cKO*^ pachytene cells across different clusters of genes group according to their expression across spermatogenesis. The expression profile of each cluster is shown. The black line indicates the mean expression of the group, and the gray area indicates the standard deviation. The number of transcripts in a cluster (n) and the number of upregulated transcripts in that cluster (x) are shown for each cluster. The *P* values (Hypergeometric test) for enrichment or depletion are also indicated. S spermatogonia stem cells, L leptotene-zygotene, P pachytene-diplotene, Sp round spermatids

Gene expression analysis revealed the deregulation of many transcripts in *Tut4/7*^*cKO*^ pachytene spermatocytes. The majority of the dysregulated genes were upregulated (732 genes) in comparison to genes whose expression was downregulated (125 genes), which is an expectation of removing an RNA degradation signal (Fig. 5a). Several representative transcripts were additionally validated by RT-qPCR (Fig. S6a). The same alteration of gene expression was observed in *Tut4/7*^*cAAD*^ as in *Tut4/7*^*cKO*^ pachytene spermatocytes indicating that TUT4/7-mediated uridylation underlies these changes in the transcriptome (Fig. S6b). Gene ontology analysis of the deregulated genes did not identify any germline-, spermatogenesis-, meiosis-specific processes but rather revealed generic macromolecule metabolic pathways (Fig. S6c). We next analyzed mRNA poly(A) tail length and 3′ uridylation from the transcriptomes of *Tut4/7*^*CTL*^ and *Tut4/7*^*cKO*^ pachytene spermatocytes using TAIL-seq. This analysis revealed that the mode length of the poly(A) tails in *Tut4/7*^*CTL*^ pachytene spermatocytes was 55 nucleotides and remained constant for transcripts that are not upregulated in the *Tut4/7*^*cKO*^ pachytene cells. Loss of TUT4/7 does not grossly alter the poly(A) tail length in the not upregulated genes whereas the poly(A) tails of the upregulated genes become extended (*P* < 0.05) (Fig. 5b). Deficiency of TUT4/7 reduced terminal uridylation of genes not upregulated in the *Tut4/7*^*cKO*^ pachytene spermatocytes by 5.8-fold whereas the uridylation of upregulated transcripts was absolutely dependent upon TUT4/7 (Fig. 5c). The loss of TUT4/7 also affects the terminal uridylation of histone mRNAs that do not have poly(A) tails (Fig. S6d). To understand if TUT4/7-deficiency results in alterations to the proteome, we performed mass spectrometry analysis of protein extracts from pachytene cells (Fig. S6e). This analysis revealed that TUT4/7*-*deficiency resulted in overall changes to the proteome of pachytene cells with some upregulated transcripts in *Tut4/7*^*cKO*^ pachytene cells also being upregulated at the protein level (Fig. S6e).

We next sought to explore the relationship between the deregulated genes in the *Tut4/7*^*cKO*^ pachytene cells and spermatogenesis. We thus addressed if we could identify enrichment or depletion of the TUT4/7-dependent upregulated genes in any of the spermatogenesis gene expression patterns identified in Fig. 1b. We found the highest significance in enrichment for cluster 2 (*P* < 10^−10^), the large gene set that comprises the transcripts that are downregulated in the transition from zygotene to pachytene (Fig. 5d). Significance was also found in other clusters that also showed downregulation in pachytene. Conversely, depletion of the upregulated gene set was observed in clusters where genes were upregulated or remained unchanged across spermatogenesis (Fig. 5d). We also identified enrichment and depletion in the equivalent gene expression clusters (Fig. S7) identified from the analysis of the spermatogenic single-cell dataset^49^ (Fig. S1). In essence, TUT4/7 and their uridylation activities are required to degrade transcripts in pachytene spermatocytes, and the failure to do so derails spermatogenesis.

### Long 3′ UTRs and AU elements are features of TUT4/7-regulated transcripts in the male and female germline

**Fig. 6:**
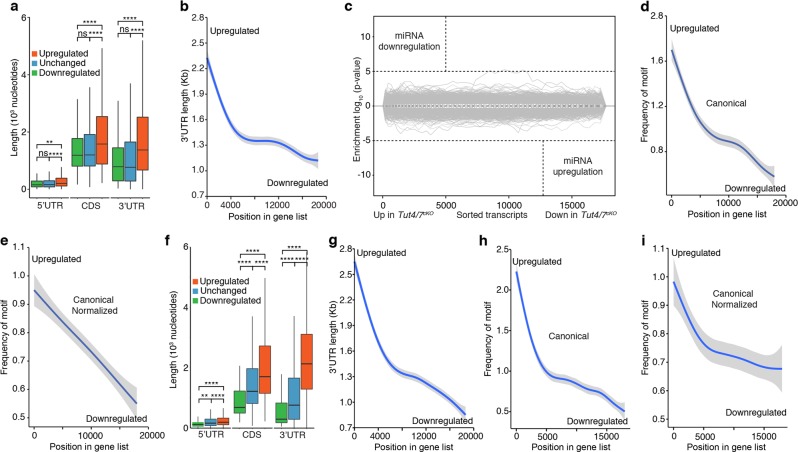
TUT4/7 target transcripts in pachytene spermatocytes and GV oocytes that are enriched for AU-rich elements in long 3′ UTRs. **a** Box plot of 5′UTR, CDS and 3′UTR length for downregulated, unchanged and upregulated transcripts in *Tut4/7*^*cKO*^ pachytene cells. The center value represents the mean length, and the upper and middle hinges the first and third quartiles, respectively. (ns not significant, **P < 0.01, ****P < 0.0001, Wilcoxon test two-sided). **b** GAM fit of 3′UTR length to the ranked position of genes according to their differential expression in *Tut4/7*^*cKO*^ pachytene cells. Genes are ranked from upregulated to downregulated. **c** Sylamer analysis of miRNA signatures for transcripts ranked according to changes in expression between *Tut4/7*^*CTL*^ and *Tut4/7*^*cKO*^ pachytene cells. **d** GAM fit of the frequency of the canonical AU-rich element AUUUA to transcripts ranked according to changes in expression between *Tut4/7*^*CTL*^ and *Tut4/7*^*cKO*^ pachytene cells. **e** GAM fit as in **d** where the frequency of the motif is normalized to the length of the 3′UTR. **f** Box plot of 5′UTR, CDS and 3′UTR length for downregulated, unchanged and upregulated transcripts in *Tut4/7*^*cKO*^ GV oocytes. The center value represents the mean length, and the upper and middle hinges the first and third quartiles, respectively. (**P < 0.01, ****P < 0.0001, Wilcoxon test two-sided). **g** GAM fit of 3′UTR length to the ranked gene position according to differential expression in *Tut4/7*^*cKO*^ GV oocytes. Genes are ranked from upregulated to downregulated. **h** GAM fit of the frequency of the canonical AU-rich element AUUUA to transcripts ranked according to changes in expression between *Tut4/7*^*CTL*^ and *Tut4/7*^*cKO*^ GV oocytes. **i** GAM fit as in **h** where the frequency of the motif is normalized to the length of the 3′UTR

Next, we sought to understand which features of TUT4/7-regulated transcripts could identify them for degradation in pachytene spermatocytes. Analysis of transcripts revealed that 5′UTR, CDS and 3′UTR were increased in length in transcripts that are upregulated in *Tut4/7*^*cKO*^ pachytene spermatocytes (Fig. 6a). 3′UTRs contain many types of regulatory signals ranging from consensus sequences for RNA binding proteins and miRNA binding sites to the actual length of the UTR itself.^57,58^ TUT4/7-upregulated transcripts have 3′UTRs with a mean length 500 nucleotides longer than the UTRs of transcripts that are not upregulated (Fig. 6a, b). Transcripts with long 3′UTRs have been shown to be less stable because they recruit more UPF complex that results in transcripts being degraded by the NMD pathway.^58^ Since *Upf2*^*cKO*^ (*Upf2*^*Fl/Fl*^*; Stra8Cre Tg*^*+*^) has a similar spermatogenic phenotype to *Tut4/7*^*cKO*^ mice,^59^ we speculated that there could be involvement in the same long 3′UTR-dependent degradation pathway. To test this hypothesis, we isolated pachytene spermatocytes from *Upf2*^*cKO*^ mice and performed gene expression analysis. Although there was an overlap in the upregulated genes between the two mutants, UPF2 primarily regulates spermatogenesis-specific transcripts that are upregulated in pachytene spermatocytes (Fig. S8). Therefore, we concluded that TUT4/7 do not target transcripts with long 3′UTR as part of the UPF pathway.

Next, we sought to understand if there are any cis-regulatory elements present in the 3′UTRs of the transcripts upregulated by *Tut4/7*^*cKO*^. While we did observe alterations to miRNA expression in *Tut4/7*^*cKO*^ pachytene spermatocytes, these were minor (Fig. 4b, c). Accordingly, Sylamer analysis^60^ did not reveal any major changes in the *Tut4/7*^*cKO*^ transcriptome that could be accounted for by miRNAs (Fig. 6c). Next, we searched for motifs in the 3′UTRs of the TUT4/7-regulated genes and found a significant enrichment for the canonical AU-rich element (ARE) (Fig. 6d). The enrichment was independent of 3′UTR length since it was still observed when ARE frequencies were normalized to the 3′UTR length (Fig. 6e).

Long 3′UTRs are also a feature of TUT4/7-regulated genes in *Tut4/7*^*cKO*^ GV oocytes (Fig. 6f, g). Deletion of *Upf2* during oocyte growth results in female infertility (Fig. S9a-b) due to the production of immature GV oocytes that are not competent to undergo meiotic maturation (Fig. S9c, d). Again, as in spermatocytes, UPF2 mainly regulates different set of transcripts than those upregulated in TUT4/7-deficient GV oocytes (Fig. S9e-g). However, enrichment for AREs was also observed in TUT4/7-regulated genes from *Tut4/7*^*cKO*^ GV oocytes (Fig. 6h, i). In summary, we found that the TUT4/7-regulated transcripts in the germline are identified by having long 3′UTRs enriched with AREs.

## Discussion

Here we show that the zygotene to pachytene transition is not only associated with the resumption of transcription but also a requirement for substantial mRNA degradation. We show that TUT4/7-mediated 3′ uridylation contributes to this pachytene wave of mRNA degradation and is essential for pachytene progression. The abundant TUT7 expression in round spermatids suggests that 3′ uridylation may be a critical regulator of spermiogenesis. The lack of phenotype in *Tut7*^*−/−*^ mice does not exclude a possible redundant function of TUT7 and TUT4 during this process. Unfortunately, the meiotic arrest in *Tut4/7*^*cKO*^ mice makes us unable to explore this possibility. Maternal TUT4/7 function is also known to be required for oocyte maturation in mice and for the maternal to zygotic transition in zebrafish.^44,48^ Here we identify an essential embryonic function for TUT4/7 in regulating growth from mid-gestation onwards. Previous studies had shown that TUT4-deficiency in mice resulted in postnatal growth retardation.^61^ Here we show that TUT4 and TUT7 are redundant during embryonic growth. While the molecular basis for this growth defect in TUT4/7-deficient embryos remains unknown, these enzymes are essential for neonatal viability. TUT7-deficiency alone does not have an impact upon animal growth,^62^ but it is important in regulating macrophages and innate immunity.^62^ Cde1, the orthologue of TUT4/7 in *C. elegans*, restricts the RNA Orsay virus through facilitating viral genome 3′ uridylation.^47^ Indeed, TUT4/7-mediated uridylation also impairs influenza virus replication in mouse and human cells.^47^ Similar to reports from studies in a variety of somatic cells,^44^ we have not observed a major function for TUT4/7 in regulating miRNA expression in pachytene spermatocytes. Likewise, the loss of TUT4/7 did not have an impact upon piRNA expression but we did find that TUT4/7 can 3′ uridylate piRNAs. The loss of uridylation does not affect piRNA function; however, this outcome is expected given that complementarity of the first 21-23 5′ nucleotides of the piRNA is required for slicing of a target transcript^19^ and also the fact that piRNA with long 3′ extensions resulting from incomplete piRNA processing in TDRKH- and PNLDC1-deficient mice retain partial function.^63–65^ Our results reveal a non-essential contribution for TUT4/7 to piRNA 3′ ends.

Our results demonstrate that TUT4/7-mediated 3′ uridylation is a key determinant of both male and female germ cell development as well as fertility. One interesting difference we observed between male and female TUT4/7-deficient transcriptomes is the failure of TUT4/7-regulated transcripts to accumulate with short poly(A) tails in pachytene spermatocytes. We speculate that upon poly(A) tail shorting in TUT4/7-deficient spermatocytes, the lack of 3′ uridylation and subsequent failure to degrade the respective transcripts allows poly(A) polymerases (PAPs) to re-lengthen the poly(A) tails. Indeed, PAPs are strictly regulated in oocytes, as poly(A) tail elongation is the principal mechanism to awaken transcripts from dormancy,^66,67^ whereas spermatocytes may have greater and less restricted PAP activity. Here we show that long 3′UTRs are a feature of TUT4/7-regulated transcripts in pachytene spermatocytes and this characteristic is also found in TUT4/7-regulated maternal transcripts. Both in spermatocytes and GV oocytes we can show that the accumulation of the UPF complex on long 3′UTRs is not the basis of the targeted mRNA degradation. However, the long 3′UTRs may accumulate a higher concentration of 3′UTRs-specific or -enriched RNA binding proteins that in turn may recruit TUT4/7 to target the transcript for uridylation upon deadenylation. Indeed TUT4/7 has been recently shown to interact with MOV10,^68^ a 3′UTR-binding RNA helicase.^69^ Information encoded within the long 3′UTRs may also identify these transcripts for programmed elimination. The miRNA pathway could merit a candidate mechanism since miRNA binding sites are predominantly located within 3′UTRs; however, the mild degree of miRNA deregulation and the lack of consequent transcriptome changes due to TUT4/7 deficiency likely excludes a major contribution of miRNA uridylation in pachytene spermatocytes. In addition, oocytes do not require an active miRNA pathway.^70,71^ Our data do reveal that enrichment for AU-rich elements in the 3′UTRs is a common feature of both spermatocyte and maternal TUT4/7-regulated transcripts. Canonical AREs are predominantly associated with RNA decay^72,73^ and could provide the decay signal for mRNA degradation in both germlines.^74–76^ In summary, we have demonstrated a common function for TUT4/7-mediated 3′ uridylation in the regulation of germline transcriptomes essential for both male and female fertility.

## Materials and methods

### Animals

All mouse alleles used in this study were previously characterized: *Tut4*^*HA-GFP*^, *Tut7*^*HA-GFP*^, *Tut4*^*fl*^, *Tut4*^*-*^, *Tut4*^*AAD*^, *Tut7*^*fl*^, and *Tut7*^*-*^ alleles were described in ref. ^44^; the *Ythdf2*^*HA-Fl*^ allele was described in ref. ^33^; the *Stra8Cre* allele was described in ref. ^51^; *Zp3Cre* allele was described in ref. ^77^; the *Mili*^*null*^ allele was described in ref. ^8^; and the *Upf2*^*fl*^ allele was described in ref. ^78^. All lines were kept in a C57BL/6 N background. Animals were maintained at the European Molecular Biology Laboratory (EMBL), Mouse Biology Unit in Monterotondo, Italy and later at the University of Edinburgh, MRC Centre for Regenerative Medicine in Edinburgh, UK in accordance to regulations of the Italian health ministry and the UK Home Office, respectively.

### Germ cell isolation

To isolate leptotene-zygotene cells, pachytene-diplotene cells, and round spermatids, we used the method developed by Bastos and colleagues.^79^ Briefly, testes from adult mice (2–6 months old) were dissected, decapsulated, and digested with Collagenase (0.05%; Sigma-Aldrich, C7657) in EKRB medium under gentle agitation at 32 °C until the seminiferous tubules were separated from each other. Subsequently, cells were trypsinized (0.05%; Sigma-Aldrich, T6763) in the presence of DNase I (0.001%; Sigma-Aldrich, DN25) also under gentle agitation at 32 °C followed by pipetting to obtain a single-cell suspension. Cells were later stained with Hoechst 33342 (10 μg/mL) and propidium iodide (PI) (2 μg/mL; Sigma-Aldrich, 81845) for 30 min at 32 °C in EKRB medium with 2% FCS. Finally, cells were FACS sorted using a FACSAria II (BD Biosciences) according to their characteristic blue Hoechst fluorescence (450/50 filter) and red Hoechst fluorescence (670/30 filter).

To determine the purity of the round spermatid population, the nuclear morphology of the Hoechst-stained sorted population was analyzed by fluorescence microscopy. The population purity was estimated to be of 90% or more. The purity of the spermatocyte populations was previously determined to be of 85% by SCP3 staining.^8^ Pachytene-diplotene cells in wild-type samples had ~10% of polynucleated round spermatids contamination whereas isolated *Tut4/7*^*cKO*^ pachytene-diplotene cells had a contamination of ~1%. To minimize differences in gene expression associated with round spermatids transcripts, for control samples, we made use of a *Ythdf2*^*HA-Fl*^ allele that expresses a GFP-YTHDF2 fusion protein at high levels in pachytene-diplotene cells. In this case, cells sorted from *Ythdf2*^*HA-Fl/+*^ animals were additionally selected for GFP-high expression. Pachytene-diplotene cells resulting from this sorting strategy had a 1% contamination of polynucleated round spermatids. Spermatogonial stem cells were cultured as described by Kanatsu-Shinohara and colleagues.^80^

For the collection of GV oocytes, 3–5 weeks old females were injected with 5 IU of pregnant mare serum gonadotropin (PMSG) (Henry Schein). GV oocytes were collected by puncturing the ovarian follicles with an injection needle in M2 media (Sigma-Aldrich) 44–48 h after PMSG injection. Subsequently, GV oocytes were released from the somatic cells via manual mechanical separation.

For the collection of MII oocytes, 3–6 weeks old females were injected with 5 IU of PMSG and after 46–48 h with chorionic gonadotropin (hCG) (Intervet). MII oocytes were isolated from the oviduct of the hormone-stimulated females 14 h after the hCG injection. mMII oocytes were cleaned from the somatic cells with hyaluronidase (Sigma-Aldrich) in M2 media.

### Bioinformatics

Microarray data were analyzed using the limma package.^81^ Intensities were normalized using the rma function; a linear model was then fitted with lmFit and, differential expression was assessed using the eBayes function. For GV analyses, *Tut4/7*^*cKO*^ and control samples were obtained from ref. ^44^. For clustering analyses, microarray data was generated in this study for SSCs, leptotene/zygotene spermatocyte, and round spermatids populations and a published dataset was used for pachytene/diplotene population.^24^ To cluster genes according to changes in gene expression across different stages of spermatogenesis, we used the Markov clustering algorithm (MCL) implemented in the BioLayout Express application.^82^ Publicly available single-cell RNA sequencing data^49^ was also used for cluster generation. In this case, read counts were summed for each of the following cell types: type A1 spermatogonia, S phase type B spermatogonia, G2/M phase type B spermatogonia, leptotene spermatocytes, zygotene spermatocytes, early-pachytene spermatocytes, mid-pachytene spermatocytes, late-pachytene spermatocytes, diplotene spermatocytes, metaphase I cells, steps 1-2 round spermatids, steps 3-4 round spermatids, steps 5-6 round spermatids, and steps 7-8 round spermatids. Summed read counts across the different cell types were then normalized using DESeq2^83^ and used to generate clusters also with MCL.^82^ For visualization purposes, we plotted the mean of the normalized read count for different cell groups as follows: type A1 spermatogonia, S phase type B spermatogonia and G2/M phase type B spermatogonia (G); leptotene and zygotene spermatocytes (L/Z); early- and mid-pachytene spermatocytes (eP); late-pachytene spermatocytes, diplotene spermatocytes and metaphase I cells (lP); steps 1-2 round spermatids and steps 3-4 round spermatids (RS2/4); and steps 5-6 round spermatids and steps 7-8 round spermatids (RS6/8). To evaluate the enrichment of upregulated transcripts in *Tut4/7*^*cKO*^ vs *Tut4/7*^*CTL*^ pachytene cells in different spermatogenesis expression clusters, we used the hypergeometric test. Ternary plots were generated with the ggtern package.^84^ Normalized intensities from *Tut4/7*^*CTL*^, *Tut4/7*^*cKO*^ and *Tut4/7*^*cAAD*^ Affymetrix experiments were used as input. For gene ontology analysis, the topGO R package was used.^85^ Fisher’s exact test with the classic algorithm was used to determine enrichment of the Biological Process (BP) ontology.

For the piRNA analysis, reads were trimmed from adapter sequences using cutadapt^86^ and sequentially mapped to miRNAs, ncRNAs, transposable elements and the genome using Bowtie.^87^ To evaluate piRNA levels between different conditions, the frequency of reads from all detected 24 to 36 K-mers was compared. For miRNA, the frequency of terminal modifications and expression levels was determined as previously described.^44^ Briefly, adapter trimmed small RNAs were mapped to miRNA precursors^88^ allowing for two mismatches with Chimira.^89^ The first optimal alignment output from BLASTn was used for multi-mapped reads. To assess terminal modifications, only non-templated 3′ nucleotides were regarded as post-transcriptional additions. For expression analysis, both modified and non-modified species of the same miRNA were grouped. The DESeq2 package was used to normalize read counts and determine differential expression between *Tut4/7*^*CTL*^ and *Tut4/7*^*cKO*^ miRNAs.

To identify small RNA signatures in the UTRs of differentially expressed genes between *Tut4/7*^*CTL*^ and *Tut4/7*^*cKO*^ pachytene cells we used Sylamer.^60^ For TAIL-seq analysis, the published pipeline^90^ was used correcting for poly(A) length according to spike-in recovery as in ref. ^44^. To identify histone mRNAs’ modifications, TAIL-seq 3′-end read pairs were mapped to the mouse genome using HISAT2.^91^ Reads mapping to any histone gene were selected, and the presence of 3′ end additions was determined by analyzing the occurrence of non-templated nucleotides between the histone sequence and the 3′ end adapter sequence. To determine the enrichment of the canonical AU-rich motif, exact matches of the AUUUA sequence were counted for each 3′UTR.

### Immunofluorescence

Immunofluorescence of testes sections to detect HA-TUT7 were carried out as previously described.^24^ All other stainings were performed as described in ref. ^8^. The following primary antibodies and dilutions were used; mouse monoclonal anti-HA antibody (1:100; Covance HA.11), rabbit polyclonal anti-TUT4 antibody (1:100; Proteintech, 18980-1-AP), mouse monoclonal anti-γH2AX (1:200; Abcam, ab26350), rabbit polyclonal anti-SCP3 (1:200; Novus Biologicals, NB300-231) and anti-ORF1 L1 (1:500, homemade antibody). For apoptosis analysis, testes were fixed in 4% paraformaldehyde at 4 °C overnight, embedded in paraffin and cut in 7 μm sections. Sections were stained using the In Situ Cell Death Detection Kit (Roche, 11684817910) following manufacturer’s instructions. DNA was stained using Hoechst 33342 (5 μg/mL). Images were acquired using the Leica TCS SP5 confocal microscopy.

Oocytes were fixed in 2% formaldehyde, 0.1 M Pipes, 10 mM MgCl_2_, 2.5 mM EGTA, and 2% Triton X-100 for 15 min at 37 °C. They were washed three times in 0.1% Normal Donkey Serum (NDS) (Sigma-Aldrich, D9663) and treated with blocking solution (10% NDS and 1% Triton X-100) overnight. For the analysis of nuclear configuration, GV oocytes were incubated with 5% NDS and Hoechst 33342 (5 μg/mL) for 15 min at room temperature and mounted on a glass bottom dish (Willco Wells, HBST-3522). For microtubule staining, MII oocytes were incubated with rabbit anti-beta tubulin antibody (Cell Signaling, 2146 S) (1:200) for 1 h at 37 °C. They were later washed three times with the blocking solution before incubation with donkey anti-rabbit Alexa 488 (1:1000) in 5% NDS and Hoechst 33342 (Sigma-Aldrich) for 1 h at 37 °C. MII oocytes were mounted on Teflon-coated slides (Dutscher scientific) in groups of 5 per well.

### Phenotypic characterization

For fertility analysis, *Tut4/7*^*CTL*^, *Tut4/7*^*cKO*^, and *Tut4/7*^*cAAD*^ experimental males were mated with C57BL/6 N females. Fertility was scored only for males that were able to plug. *Upf2*^*CTL*^ and *Upf2*^*cKO*^ experimental females were mated with stud males and were subsequently plug checked. Fertility was scored only for females that were plugged. In the case of the *Tut4*^*−/−*^ or *Tut7*^*−/*−^ males, we assessed fertility by counting the number of weaned pups per litters during continuous breeding.

For sperm counts, an epididymis from an adult male was dissected and punctured to empty all its content in 1 mL of DMEM inside a six-well plate. The sperm collected were then transferred to a 1.5 mL tube, diluted 1:10 in the case of the control animals before counting with a Neubauer chamber.

For embryological analysis males and females, both having at least one *Tut4*^−^ allele and one *Tut7*^−^allele were mated in the afternoon and plugs were checked the following morning. Plugged females were sacrificed at different time points (E7.5, E9.5, E10.5, E11.5, E12.5, E13.5, E14.5, E15.5, E16.5, and E17.5) and uterine horns were collected in ice-cold PBS for embryo dissection. Genotyping was done from yolk sac-extracted DNA. A Leica MZ12 stereomicroscope was used for image acquisition of E11.5 and E13.5 embryos and handheld camera for E15.5 and E17.5 embryos as well as newborns.

### Histology

For histological analysis testes and epididymis were fixed in Bouins solution at 4 °C overnight, paraffin embedded and cut in 6–7 μm sections. Testes were stained using the Periodic Acid-Schiff (PAS) method and epididymis with the Hematoxylin and Eosin (H&E) method. Embryos were fixed in 4% paraformaldehyde and paraffin embedded. Sagittal sections 7 μm thick were stained with H&E. A Leica DM6000 B microscope was used for image acquisition.

### Molecular biology

For western blotting the following primary antibodies were used at the specified dilutions: mouse monoclonal anti-HA (1:1000; Covance MMS-101P), mouse monoclonal anti-α-Tubulin (1:1000; Sigma, T9026), rabbit polyclonal anti-TUT4 (1:1000, Proteintech, 18980-1-AP), rabbit polyclonal anti-TUT7 (1:1000; a kind gift from R. Pillai), mouse monoclonal anti-AGO2 (1:1000; O’Carroll lab, clone MA2) and rabbit polyclonal anti-SMC1A (1:5000; Bethyl, A300-055A). After primary antibody incubation, the membranes were washed with 1 × PBS with 0.1% Tween-20 and incubated with anti-mouse (1:5000) or anti-rabbit (1:10000) secondary antibodies conjugated to horseradish peroxidase. Bands were detected with a ChemiDoc imager (Bio-Rad) using the ECL Western Blotting Detection Reagents (Amersham, RPN2106) according to manufacturer’s instructions.

Total RNA was extracted from different cell populations using QIAzol. For Affymetrix analysis of SSC, leptotene-zygotene, pachytene-diplotene and round spermatids, biotinylated cDNA was synthesized with the WT expression kit (Ambion) from total RNA. The cDNA was fragmented and labeled with the WT terminal Labeling and Controls Kit (Affymetrix) and then hybridized on a GeneChip Mouse Gene 2.0 ST Array for 16 h at 45 °C. Finally, the chip was washed and stained using an Affymetrix Fluidics Station 450. For Affymetrix analysis of GV oocytes, the same protocol was used with the following modifications. The Ovation Pico WTA System V2 kit (NuGEN) was used to synthesize the biotinylated cDNA and, the Encore Biotin Module (NuGEN) was used for fragmentation and labeling.

Small RNA libraries were prepared using the NEBNext® Multiplex Small RNA Library Prep Set for Illumina (Set 1) (NEB) according to manufacturer’s instructions. Briefly, 200 ng of total RNA was obtained from FACS sorted pachytene cells from adult mice (2-6 months). The RNA was 3′ and 5′ ligated, followed by cDNA synthesis and PCR amplification using 14 cycles. Three *Tut4/7*^*CTL*^ and 3 *Tut4/7*^*cKO*^ samples were multiplexed during the amplification using barcoded primers. PCR fragments were run on a 6% polyacrylamide gel, a slice of gel spanning the 130–170-nucleotide range was excised, and the amplification product was purified. Finally, samples were pooled and sequenced in one Illumina HiSeq lane in a 50-nucleotide single-read mode.

For qPCR analysis of pachytene spermatocytes transcripts, total RNA was treated with DNase I (Sigma) for 10 min at 37 °C and then purified with RNeasy columns (Qiagen) following manufacturers’ instructions. cDNA was synthesized using random hexamers with SuperScript III reverse transcriptase (Invitrogen) following manufacturer’s instructions. PCR amplification was performed in a LightCycler 480 (Roche) in the presence of SYBR Green for signal detection. Primers sequences used are: *Slc7a5*, forward 5′-CATCATTTTGCTCGGCTTCA-3′ and reverse 5′-TTGGTGCCTTCAAAGGACAA-3′; *Ccng1*, forward 5′-GCTTTGACACGGAGACATTT-3′ and reverse 5′-AAAAGCAGCTCAGTCCAACA-3′; *Lrat*, forward 5′-CAGATATGGCTCTCGGATCA-3′ and reverse 5′-CCAAGACAGCCGAAGCAAGA-3′; *Spata22*, forward 5′-GAATGATCCAGGTGTCAGTA-3′ and reverse 5′-AGGAGCCACCTCTGGATTCA-3′; *Dazl*, forward 5′-CTGCTCCAGCTTCTGGAAAT-3′ and reverse 5′-CAGTCTGTTCTCAGGGTTAA-3′; *Stat3*, forward 5′-TGAGGAGCTGCACCTGATCA-3′ and reverse 5′-GATGTTGGAGATCACCACAA-3′; *Brd4*, forward 5′-TGGTAGCCATGGCTCGAAAA-3′ and reverse 5′-AGGAGAGGACACTGTAACAA-3′; *Ythdc2*, forward 5′-TCCTGGTGTTCTGTGGACCA-3′ and reverse 5′-CACTGCTGTCATTGGGAATA-3′; *Gapdh*, forward 5′-ATGGTGAAGGTCGGTGTGAA-3′ and reverse 5′-GGGTCGTTGATGGCAACAAT-3′; *Sod1*, forward 5′-GTGCAGGGAACCATCCACTT-3′ and reverse 5′-CCATGCTGGCCTTCAGTTAA-3′; *Apeh*, forward 5′-TGGACACCCAGACAGGAAGT-3′ and reverse 5′-GGAGAACTGGGCCACCATAA-3′; *Pdcd7*, forward 5′-ATTGACCGCTGGAGGGTGAA-3′ and reverse 5′-CTTCAGCTAAGACCCCATCA-3′; *Birc2*, forward 5′-TGTTGGGAACCTGGAGATGA-3′ and reverse 5′-AACAAACTCCTGACCCTTCA-3′. The following amplification protocol was used: one incubation step of 98 °C for 30 s followed by 40 cycles of 98 °C for 10 s, 63 °C for 10 s, and 72 °C for 15 s. The ΔΔCT method^92^ was used for differential expression analysis of transcripts in *Tut4/7*^*CTL*^ and *Tut4/7*^*cKO*^ samples.

TAIL-seq libraries were prepared as previously described.^44^ Pachytene cells from adult males (2–6 month) were FACS sorted, and 1–2 μg of total RNA was used as TAIL-seq input. Two *Tut4/7*^*CTL*^ and 2 *Tut4/7*^*cKO*^ samples were multiplexed during the amplification using barcoded primers and were run in one Illumina HiSeq lane in a 100-nucleotide paired-end mode.

### Mass spectrometry (MS)

For liquid chromatography-MS/MS analysis, samples were prepared as described.^93^ Briefly, ~2 μg of peptides corresponding to 50,000 cells were separated on an EasySpray 50 cm column (Thermo Fisher) coupled to an Orbitrap Fusion Lumos (Thermo Fisher) mass spectrometer operated in DIA mode. Data was processed using Spectronaut 12 software^94^ (Biognosys) with project-specific spectral libraries using default settings as described.^95^ Condition analysis was performed using *t*-test on individual log2 ratios of peptides of a protein, resulted *P* values were corrected for multiple testing using *Q* value to control FDR level.

### Data availability

Small RNA sequencing and microarray data have been deposited in ArrayExpress and can be accessed via the following accession numbers: E-MTAB-7062, E-MTAB-7064, E-MTAB-7065, and E-MTAB-7067.

## Supplementary information

Figure S1

Figure S2

Figure S3

Figure S4

Figure S5

Figure S6

Figure S7

Figure S8

Figure S9
