# Supplementary material for: A programmed wave of uridylation-primed mRNA degradation is essential for meiotic progression and mammalian spermatogenesis
Source: Cell Res. 2019 Jan 7;29(3):221–32. doi: 10.1038/s41422-018-0128-1 (PMC6420129; doi:10.1038/s41422-018-0128-1)
Supplement: Supplementary file 1 — Figure S1 [file 41422_2018_128_MOESM1_ESM.pdf]

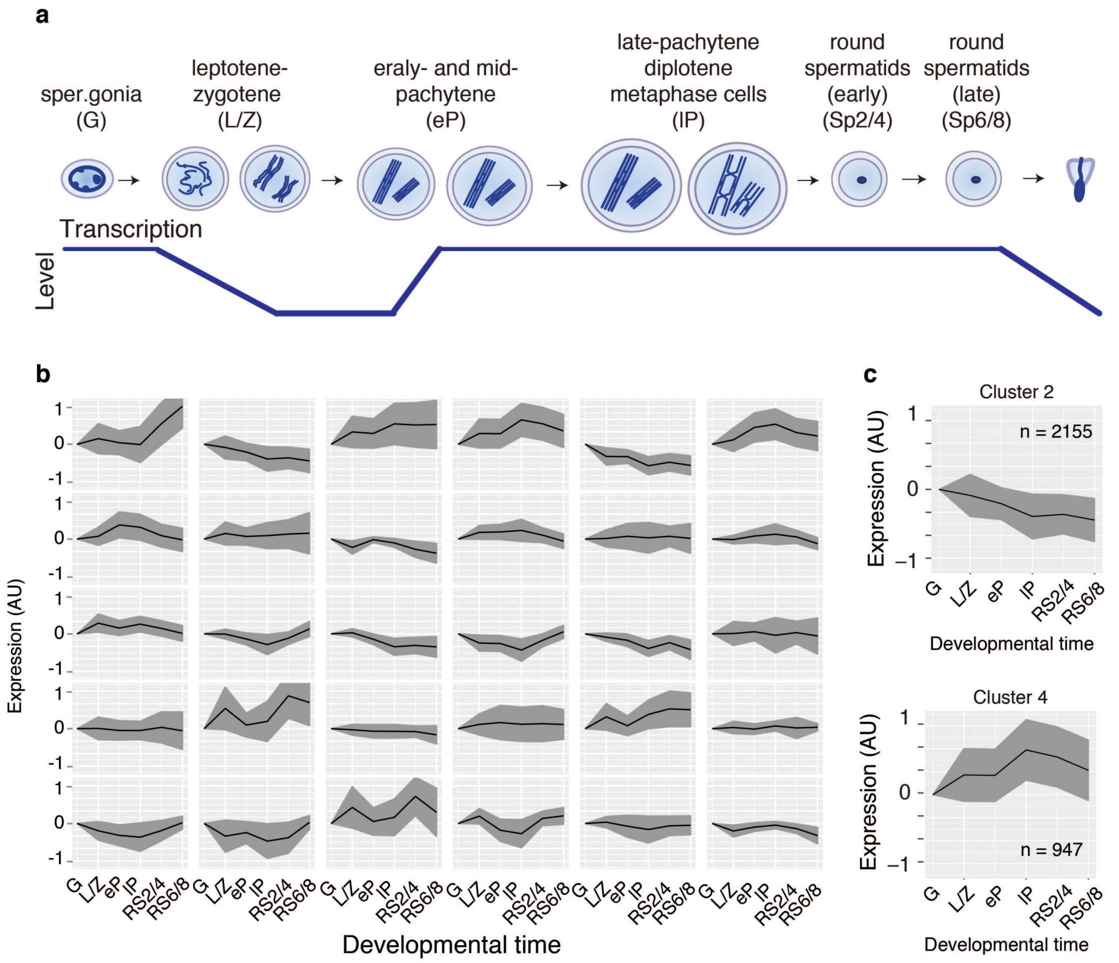

Supplementary Figure 1: Analysis of single-cell sequencing-derived spermatogenic populations reveals a programmed wave of RNA degradation during the leptotene-zygotene to pachytene transition. a, Schematic representation of different stages of spermatogenesis. Spermatogonia (G), leptotene and zygotene spermatocytes (L/Z), early- and mid-pachytene spermatocytes (eP), late-pachytene spermatocytes, diplotene spermatocytes and metaphase I cells (lP); step 1 to step 4 round spermatids (RS2/4); and step 5 to step 8 round spermatids (RS6/8) are depicted. On the bottom, levels of transcriptional activity throughout the differentiation process are indicated. **b**, Expression pattern across spermatogenesis of the top 30 gene clusters identified using the Markov clustering algorithm. The black line shows the transcripts mean expression and the grey area indicates the standard deviation. Different cell types are grouped as indicated in **a**. **c.** Examples of prominent clusters with

different expression patterns are shown. n, number of transcripts in the cluster. Different cell types are grouped as indicated in **a**.
