# Supplementary material for: A programmed wave of uridylation-primed mRNA degradation is essential for meiotic progression and mammalian spermatogenesis
Source: Cell Res. 2019 Jan 7;29(3):221–32. doi: 10.1038/s41422-018-0128-1 (PMC6420129; doi:10.1038/s41422-018-0128-1)
Supplement: Supplementary file 2 — Figure S2 [file 41422_2018_128_MOESM2_ESM.pdf]

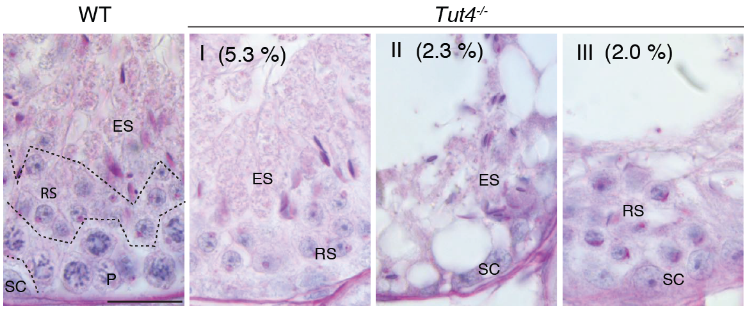

Supplementary Figure 2. *Tut4$^{-/-}$* testes show a low frequency of aberrant seminiferous tubules. Micrographs of PAS-stained sections from WT and *Tut4$^{-/-}$* mice seminiferous tubules. Panel I, tubules without primary spermatocytes. Panel II, tubules with Sertoli cells and some elongated spermatids. Panel III, tubules with other aberrant morphologies. The frequency of the different types of aberrant tubules is indicated in parenthesis. Different cell types are indicated: ES, elongated spermatids; RS, round spermatids; P, pachytene cells; SC, Sertoli cells. Scale bar, 20 μm.
