# Supplementary material for: A programmed wave of uridylation-primed mRNA degradation is essential for meiotic progression and mammalian spermatogenesis
Source: Cell Res. 2019 Jan 7;29(3):221–32. doi: 10.1038/s41422-018-0128-1 (PMC6420129; doi:10.1038/s41422-018-0128-1)
Supplement: Supplementary file 3 — Figure S3 [file 41422_2018_128_MOESM3_ESM.pdf]

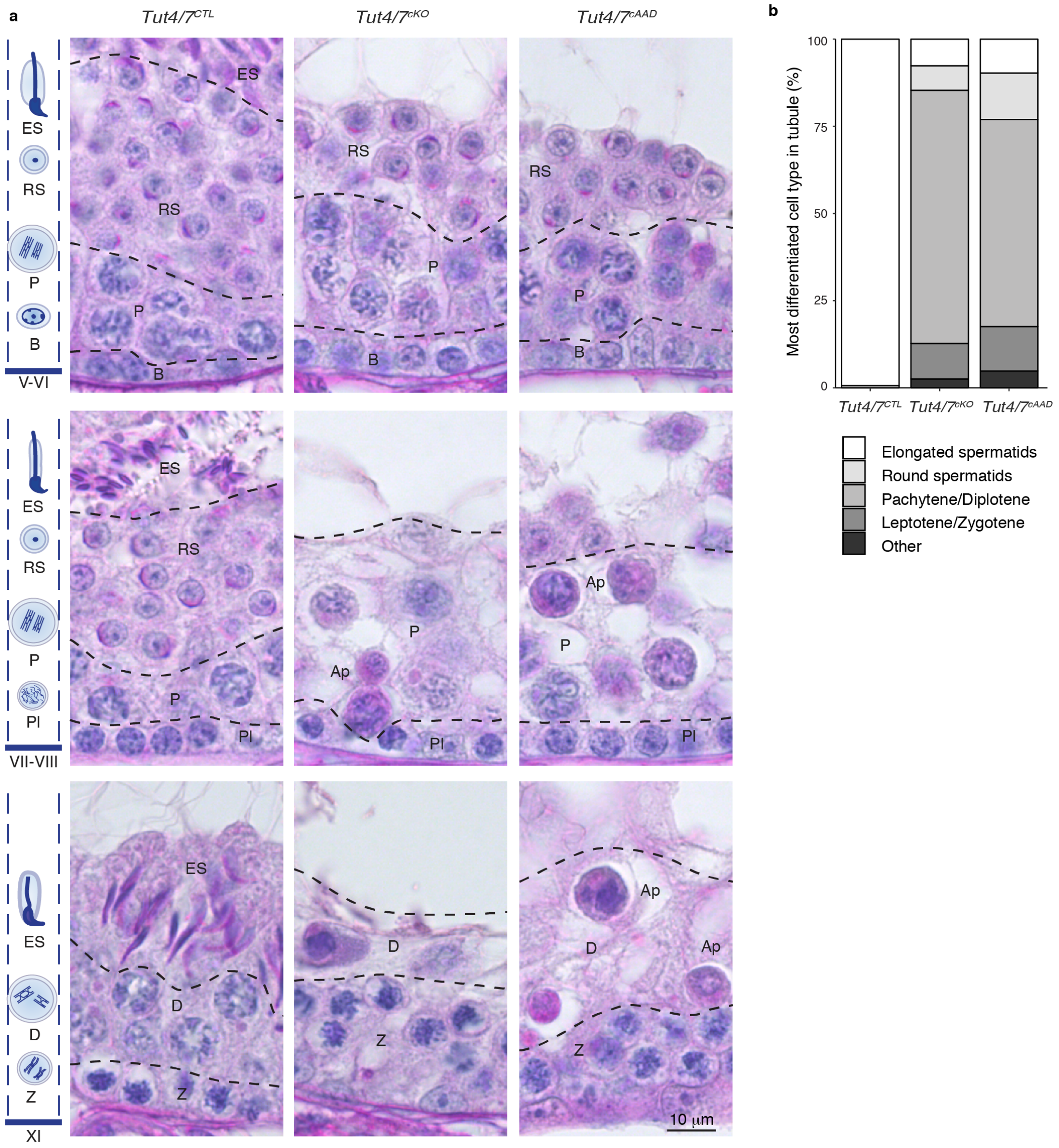

Supplementary Figure 3. TUT4/7-deficiency results in late pachytene spermatogenic arrest. a. PAS staining of tubules at stages V-VI, VII-VIII or XI of the seminiferous cycle from *Tut4/7*^{CTL}, *Tut4/7*^{cKO} and *Tut4/7*^{cAAD} animals. On the left, a schematic representation of the cell layers present at the different stages is presented. ES, elongating spermatids; RS, round spermatids; P, pachytene cell; B, B spermatogonia; Pl, pre-leptotene cell; D, diplotene cell; Z, zygotene cell and Ap, apoptotic cell. **b.** The percentages of most differentiated cell type across seminiferous tubules from *Tut4/7*^{CTL}, *Tut4/7*^{cKO} and *Tut4/7*^{cAAD} animals are shown. (Animals per genotype, n=3; Tubules per animal, n≥50.)
