# Supplementary material for: A programmed wave of uridylation-primed mRNA degradation is essential for meiotic progression and mammalian spermatogenesis
Source: Cell Res. 2019 Jan 7;29(3):221–32. doi: 10.1038/s41422-018-0128-1 (PMC6420129; doi:10.1038/s41422-018-0128-1)
Supplement: Supplementary file 4 — Figure S4 [file 41422_2018_128_MOESM4_ESM.pdf]

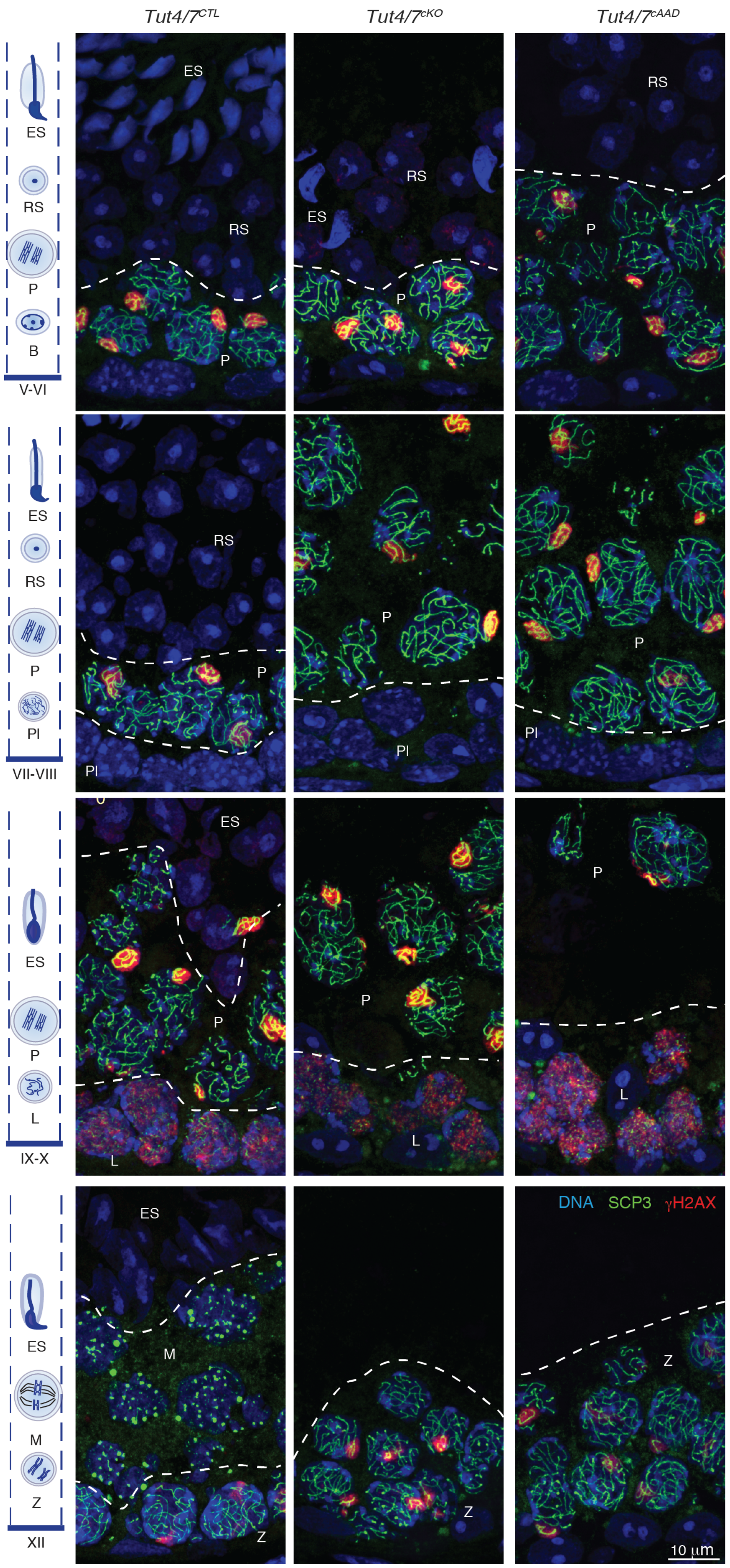

Tut4/7CTL
Tut4/7cKO
Tut4/7cAAD
ES
RS
P
B
V-VI
Pl
VII-VIII
L
IX-X
M
Z
XII
DNA
SCP3
γH2AX
10 μm

Supplementary Figure 4. TUT4/7 are required for meiotic progression. Micrographs of seminiferous tubules from *Tut4/7*^{CTL}, *Tut4/7*^{cKO} and *Tut4/7*^{cAAD} animals stained for SCP3 (green), γH2AX (red) and DNA (blue) are shown. Tubules from stages V-VI, VII-VIII, IX-X and XII are presented for the indicated genotypes. Dashed lines delimit cells layers. On the left, schematic representations of the stages are depicted. (ES, elongated spermatid; RS, round spermatid; P, pachytene spermatocyte; Pl, pre-leptotene spermatocyte, L, leptotene cell; M, metaphase I cell; Z, zygotene spermatocyte). Scale bar, 10 μm.
