# Supplementary material for: A programmed wave of uridylation-primed mRNA degradation is essential for meiotic progression and mammalian spermatogenesis
Source: Cell Res. 2019 Jan 7;29(3):221–32. doi: 10.1038/s41422-018-0128-1 (PMC6420129; doi:10.1038/s41422-018-0128-1)
Supplement: Supplementary file 5 — Figure S5 [file 41422_2018_128_MOESM5_ESM.pdf]

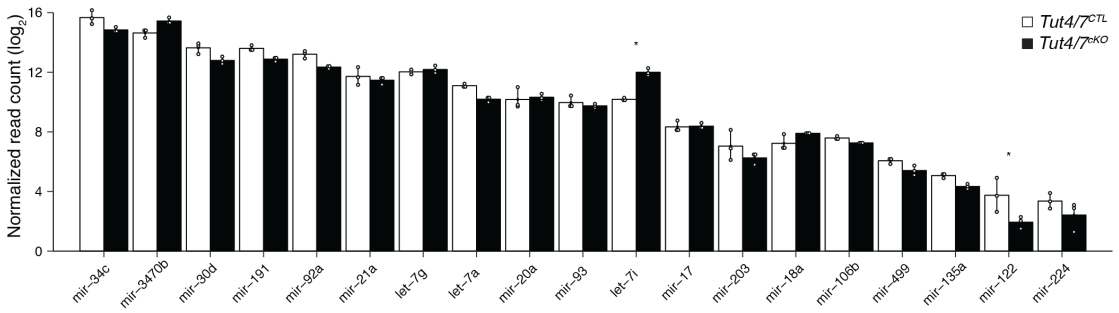

Supplementary Figure 5. miRNA expression in *Tut4/7^{CTL}* and *Tut4/7^{cKO}* pachytene spermatocytes. Normalized read count of the indicated miRNAs in *Tut4/7*^{CTL} (white) and *Tut4/7*^{cKO} (black) spermatocytes is presented. The height of the bar indicates the mean read count and the error bar the read count range. Each dot represents a biological replicate. miRNAs significantly changing more than two-fold ($P < 0.01$) are indicated with a star.
