# Supplementary material for: A programmed wave of uridylation-primed mRNA degradation is essential for meiotic progression and mammalian spermatogenesis
Source: Cell Res. 2019 Jan 7;29(3):221–32. doi: 10.1038/s41422-018-0128-1 (PMC6420129; doi:10.1038/s41422-018-0128-1)
Supplement: Supplementary file 6 — Figure S6 [file 41422_2018_128_MOESM6_ESM.pdf]

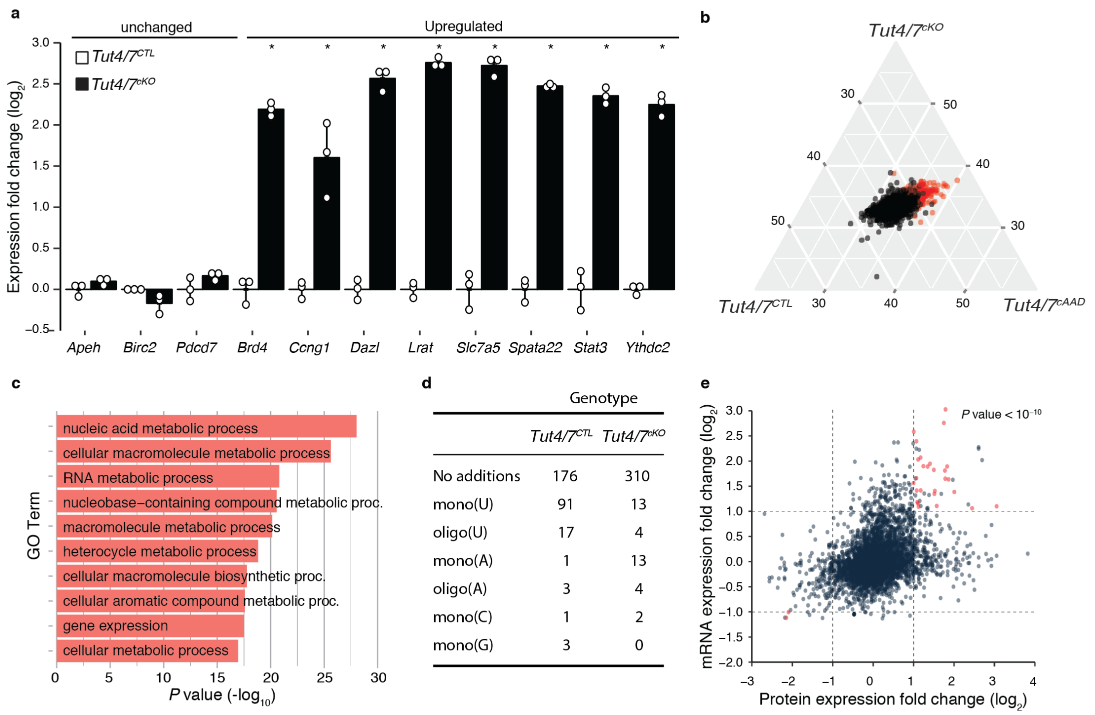

	Genotype	
	Tut4/7CTL	Tut4/7cKO
No additions	176	310
mono(U)	91	13
oligo(U)	17	4
mono(A)	1	13
oligo(A)	3	4
mono(C)	1	2
mono(G)	3	0

Supplementary Figure 6. The uridylation activity of TUT4/7 is required for mRNA downregulation. **a**, qPCR analysis of different transcripts in *Tut4/7*^{CTL} (white) vs *Tut4/7*^{cKO} (black) pachytene spermatocytes is shown. The height of the bars represents the mean fold change, and the error bars the range. Each dot represents a biological replicate. Transcripts upregulated or unchanged in the array profiling are indicated. mRNAs that significantly change more than two-fold (P < 0.01) are indicated with a star. **b**, Ternary plot of mRNA expression levels from *Tut4/7*^{CTL}, *Tut4/7*^{cKO} and *Tut4/7*^{cAAD} pachytene cells. Upregulated transcripts in *Tut4/7*^{cKO} and *Tut4/7*^{cAAD} samples are highlighted in red (P < 0.05 and fold-change > 2, moderated t-statistic adjusted; *Tut4/7*^{CTL}, n=4; *Tut4/7*^{cKO}, n=3; *Tut4/7*^{cAAD}, n=4). **c**, Gene ontology analysis of upregulated transcripts in TUT4/7-deficient pachytene cells. The enrichment for each term is shown. **d**, Number of histone transcripts with 3' end terminal additions in *Tut4/7*^{CTL} and *Tut4/7*^{cKO} pachytene spermatocytes. **e**, Scatter plot of mRNA vs protein expression fold change between *Tut4/7*^{CTL} and *TUT4/7*^{cKO} pachytene spermatocytes. Genes with proteins and transcript simultaneously changing (adjusted P < 0.01) more than two-fold are highlighted in red. The sensitivity of our mass spectrometry analysis allowed us to identify a total of 3604 proteins.

177 out of the 732 genes upregulated at the transcript level were detected. While only 1 in 40 genes with not upregulated transcripts show upregulation at the protein level, the ratio increases to 1 in 6 for genes with upregulated transcripts. The significance of the protein and transcript upregulation dependency evaluated using a Pearson's Chi-squared test is indicated at the panel top.
