# Supplementary material for: A programmed wave of uridylation-primed mRNA degradation is essential for meiotic progression and mammalian spermatogenesis
Source: Cell Res. 2019 Jan 7;29(3):221–32. doi: 10.1038/s41422-018-0128-1 (PMC6420129; doi:10.1038/s41422-018-0128-1)
Supplement: Supplementary file 7 — Figure S7 [file 41422_2018_128_MOESM7_ESM.pdf]

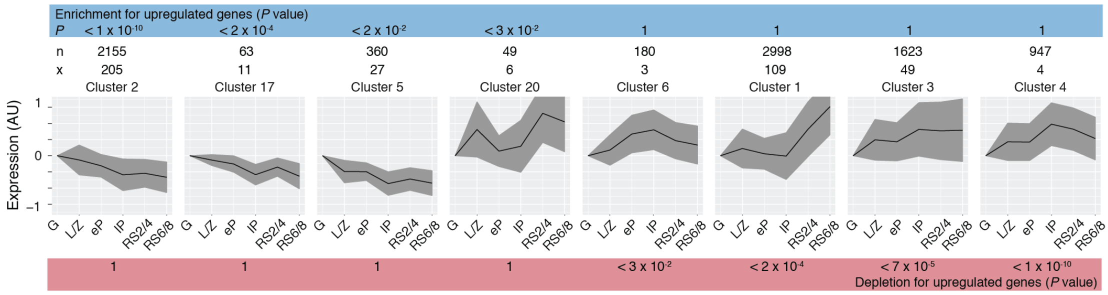

Supplementary Figure 7. Enrichment analysis of *Tut4/7^{cKO}* upregulated transcripts in different mRNA clusters defined according to their expression patterns across spermatogenesis. The significance of the enrichment (top) or depletion (bottom) of the upregulated genes in each cluster is indicated together with the size of the cluster (n) and the number of upregulated genes in the clusters (x). The expression pattern is depicted for each cluster. The black line indicates the mean expression levels of all the genes in the cluster and the gray area the standard deviation. The clusters were generated using single-cell level expression profiles from; type A1 spermatogonia, S phase type B spermatogonia and G2/M phase type B spermatogonia (G); Leptotene and zygotene spermatocytes (L/Z); early- and mid- pachytene spermatocytes (eP); late-pachytene spermatocytes, diplotene spermatocytes and metaphase I cells (lP); steps 1-2 round spermatids and steps 3-4 round spermatids (RS2/4); and steps 5-6 round spermatids and steps 7-8 round spermatids (RS6/8).
