# Supplementary material for: A programmed wave of uridylation-primed mRNA degradation is essential for meiotic progression and mammalian spermatogenesis
Source: Cell Res. 2019 Jan 7;29(3):221–32. doi: 10.1038/s41422-018-0128-1 (PMC6420129; doi:10.1038/s41422-018-0128-1)
Supplement: Supplementary file 8 — Figure S8 [file 41422_2018_128_MOESM8_ESM.pdf]

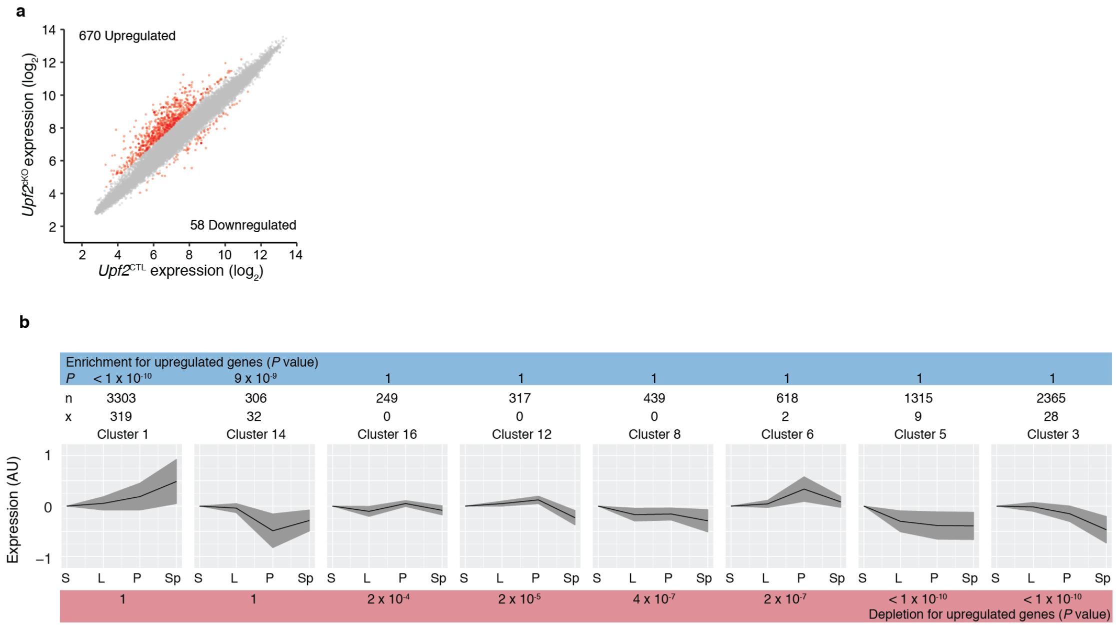

Supplementary Figure 8. UPF2 downregulates actively transcribed mRNAs in pachytene. a, Scatter plot of pachytene mRNA expression levels in *Upf2^{CTL}* vs *Upf2^{cKO}* animals. Transcripts significantly changed (P < 0.05 and fold-change > 2, moderated t-statistic adjusted; *Upf2^{CTL}*, n=4; *Upf2^{cKO}*, n=3) are highlighted in red. The numbers of significantly upregulated and downregulated genes are indicated. **b**, Enrichment analysis of upregulated transcripts in *Upf2^{cKO}* pachytene cells across different clusters of genes grouped according to their expression across spermatogenesis. The expression profile of each cluster is shown. The black line indicates the mean expression of the group, and the gray area indicates the standard deviation. The number of transcripts in a cluster (n) and the number of upregulated transcripts in that cluster (x) are shown for each cluster. The P values (Hypergeometric test) for enrichment or depletion are also indicated. (S, spermatogonial stem cells; L, leptotene-zygotene; P, pachytene-diplotene; and Sp, round spermatids).
