# Supplementary material for: A programmed wave of uridylation-primed mRNA degradation is essential for meiotic progression and mammalian spermatogenesis
Source: Cell Res. 2019 Jan 7;29(3):221–32. doi: 10.1038/s41422-018-0128-1 (PMC6420129; doi:10.1038/s41422-018-0128-1)
Supplement: Supplementary file 9 — Figure S9 [file 41422_2018_128_MOESM9_ESM.pdf]

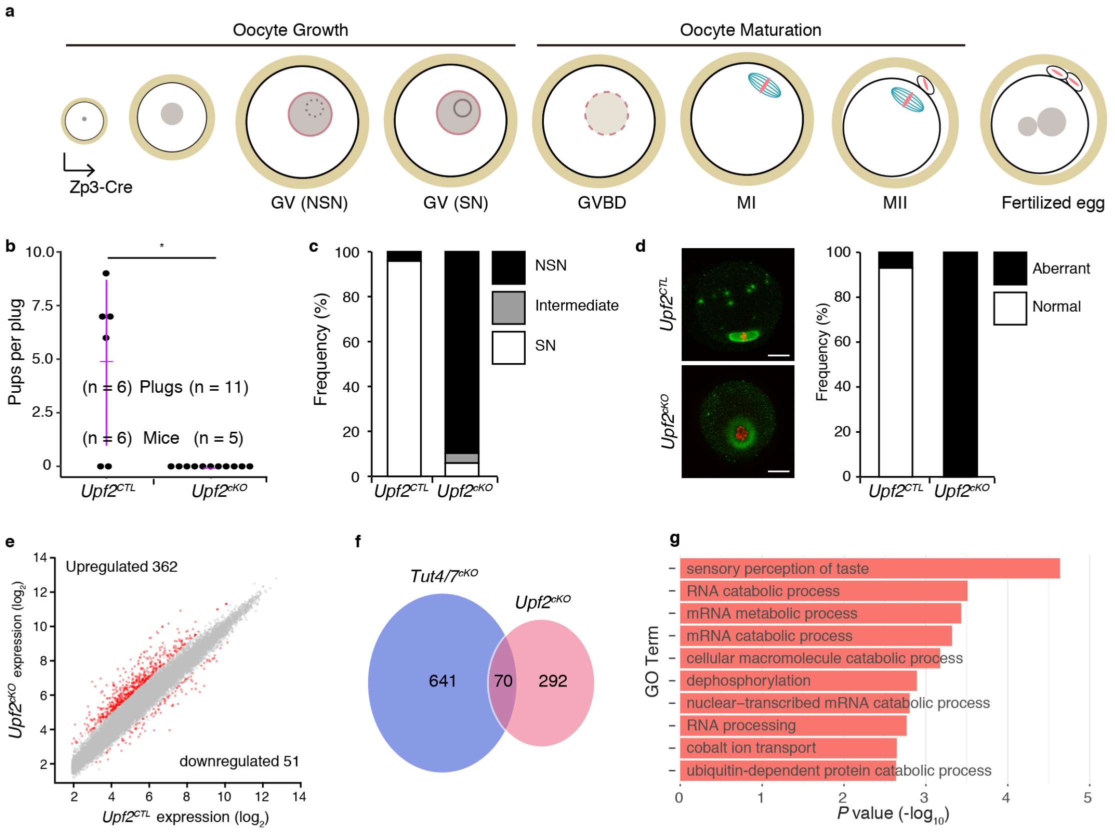


**Supplementary Figure 9. UPF2 is essential for oocyte growth. a**, Schematic representation of oocyte growth and maturation. *Zp3* starts to be expressed at the early stage of growing oocyte in primordial follicles. **b**, Number of pups born per plug from *Upf2$^{CTL}$* (*Upf2$^{+/fl}$*; *Zp3Cre Tg$^{+}$*) and *Upf2$^{cKO}$* (*Upf2$^{fl/fl}$*; *Zp3Cre Tg$^{+}$*) mice. The number of mice and plugs examined are indicated. Horizontal bars represent the mean, and the error bars the standard deviation. (**P* < 0.05; t-test two-sided). **c**, Frequency of non-surrounded nucleolus and surrounded nucleolus states in *Upf2$^{CTL}$* and *Upf2$^{cKO}$* GV oocytes. (**P* < 0.05, Fisher's exact test. *Upf2$^{CTL}$*, *n* = 74 oocytes, *n* = 3 mice; and *Upf2$^{cKO}$*, *n* = 68 oocytes, *n* = 3 mice). **d**, Left, representative confocal immunofluorescence micrographs showing *Upf2$^{CTL}$* (top) and abnormal *Upf2$^{cKO}$* (bottom) MII oocytes stained with anti-tubulin antibody (green) and Hoechst 33342 (red). Scale bars, 20 µm. Right, the frequency of abnormal MII oocytes is presented for the respective genotypes. (**P* < 0.05, Fisher's exact test. *Upf2$^{CTL}$*, *n* = 43 oocytes, *n* = 2 mice; *Upf2$^{cKO}$*, *n* = 21 oocytes, *n* = 2 mice). **e**, Expression scatter plot showing relative average expression of transcripts between control and *Upf2$^{cKO}$* GV oocytes. Significantly deregulated (*P* < 0.01, moderated *t*-statistic adjusted) genes with a fold change greater than two

are highlighted in red. (*Upf2*$^{CTL}$, *n* = 3; *Upf2*$^{cKO}$, *n* = 3). **f**, Venn diagram showing the common genes upregulated in *Tut4/7*$^{cKO}$ (*Tut4*$^{fl/fl}$; *Tut7*$^{fl/fl}$; *Zp3Cre Tg*$^{+}$) and *Upf2*$^{cKO}$ GV oocytes. **g**, Gene ontology analysis of upregulated genes in *Upf2*$^{cKO}$ GV oocytes.
